# Modelling the Force of Infection for Hepatitis A in an Urban Population-Based Survey: A Comparison of Transmission Patterns in Brazilian Macro-Regions

**DOI:** 10.1371/journal.pone.0094622

**Published:** 2014-05-20

**Authors:** Ricardo Arraes de Alencar Ximenes, Celina Maria Turchi Martelli, Marcos Amaku, Ana Marli C. Sartori, Patricia Coelho de Soárez, Hillegonda Maria Dutilh Novaes, Leila Maria Moreira Beltrão Pereira, Regina Célia Moreira, Gerusa Maria Figueiredo, Raymundo Soares de Azevedo, for the Hepatitis Study Group

**Affiliations:** 1 School of Medical Sciences, State University of Pernambuco, Recife, Pernambuco, Brazil; 2 Department of Tropical Medicine, Federal University of Pernambuco, Recife, Pernambuco, Brazil; 3 Institute of Tropical Pathology and Public Health, Department of Community Health, Federal University of Goias, Goiania, Goias, Brazil; 4 Department of Preventive Veterinary Medicine and Animal Health, School of Veterinary Medicine, University of Sao Paulo, Sao Paulo, Brazil; 5 Department of Infectious and Parasitic Diseases, School of Medicine, University of Sao Paulo, São Paulo, Brazil; 6 Department of Preventive Medicine, School of Medicine, Sao Paulo University, Sao Paulo, Brazil; 7 Liver Institute of Pernambuco – IFP, Recife, Pernambuco, Brazil; 8 Virology Centre, Adolfo Lutz Institute, Sao Paulo, Brazil; 9 Institute of Tropical Medicine, School of Medicine, Sao Paulo University, Sao Paulo, Brazil; 10 Department of Pathology, School of Medicine, Sao Paulo University, Sao Paulo, Brazil; University of North Carolina School of Medicine, United States of America

## Abstract

**Background:**

This study aimed to identify the transmission pattern of hepatitis A (HA) infection based on a primary dataset from the Brazilian National Hepatitis Survey in a pre-vaccination context. The national survey conducted in urban areas disclosed two epidemiological scenarios with low and intermediate HA endemicity.

**Methods:**

A catalytic model of HA transmission was built based on a national seroprevalence survey (2005 to 2009). The seroprevalence data from 7,062 individuals aged 5–69 years from all the Brazilian macro-regions were included. We built up three models: fully homogeneous mixing model, with constant contact pattern; the highly assortative model and the highly assortative model with the additional component accounting for contacts with infected food/water. Curves of prevalence, force of infection (FOI) and the number of new infections with 99% confidence intervals (CIs) were compared between the intermediate (North, Northeast, Midwest and Federal District) and low (South and Southeast) endemicity areas. A contour plot was also constructed.

**Results:**

The anti- HAV IgG seroprevalence was 68.8% (95% CI, 64.8%–72.5%) and 33.7% (95% CI, 32.4%–35.1%) for the intermediate and low endemicity areas, respectively, according to the field data analysis. The models showed that a higher force of infection was identified in the 10- to 19-year-old age cohort (∼9,000 infected individuals per year per 100,000 susceptible persons) in the intermediate endemicity area, whereas a higher force of infection occurred in the 15- to 29-year-old age cohort (∼6,000 infected individuals per year per 100,000 susceptible persons) for the other macro-regions.

**Conclusion:**

Our findings support the shift of Brazil toward intermediate and low endemicity levels with the shift of the risk of infection to older age groups. These estimates of HA force of infection stratified by age and endemicity levels are useful information to characterize the pre-vaccination scenario in Brazil.

## Introduction

The World Health Organisation (WHO) estimated approximately 126 million clinical cases of hepatitis A (HA) worldwide in 2005 [1]. Hepatitis A virus is mainly transmitted via the faecal/oral route by either ingestion of contaminated food or water or direct contact with an infected person [1]. Jaundice is one of the most characteristic clinical signs of the disease and can be accompanied by malaise, fatigue, anorexia and abdominal pain. There is a diversity of age patterns in the disease distribution and severity of illness, closely related to socioeconomic conditions. Viral exposure at an early age usually elicits an asymptomatic or self-limiting liver infection, with resulting life-long protective immunity. In contrast, later viral exposure occurring in adolescents and adults, which takes place in more developed settings, may cause symptomatic acute cases with increased severity and higher disease burden [1], [2]. Improving sanitation and living conditions can reduce HA infection rates, but an effective HA vaccine has also been available as a control strategy since the late 1990s [3], [4].

The vaccination policy against the HA virus (HAV) varies according to epidemiologic context. The World Health Organization (WHO) recommends universal vaccination against hepatitis A in countries with intermediate and low endemicity after cost-effectiveness analysis [1]. In South America, Argentina has implemented one-dose routine childhood vaccination with impact in the incidence of the disease [3].

Since the 90's seroprevalence studies have suggested epidemiological shift of HAV infection from high towards intermediate endemicity levels in Latin American [5], [6]. Nevertheless, prevalence close to 90% among adolescent and adults were found in and isolated communities in the North and Central West parts of Brazil [7]–[10].

In Brazil, HAV vaccination is recommended only for patients at risk of severe illness, such as those with chronic liver diseases, coagulopathy, haemoglobinpathy, cystic fibrosis, persons aged ≤13 years with HIV infection and carriers of Hepatitis B and C virus [11]. The first National Hepatitis Survey (2005–2009) conducted in a representative sample of individuals aged 5–19 years revealed the seroprevalence of HA infection to be approximately 40% in Northeast and Midwest macro-regions of Brazil [12]. The results of the survey showed robust evidence of a transition from high to lower endemicity nationwide. In the context of this transition, the highest prevalence of viral exposure, and consequently the highest incidence of HA, tended to shift from children to older age groups before the introduction of routine HA vaccinations. At the population level, the rising proportion of susceptible adults increases the severity and economic burden of the illness [13], [14].

Incidence estimates and the ratio of asymptomatic to symptomatic infection by age group have important public health implications in understanding HA transmission [15]. In addition, estimates of the incidence rates of HA by age group over time are essential parameters for evaluating the impact of interventions caused by sanitation improvement and/or vaccination implementation [16], [17]. In Brazil, a few HA incidence studies using serosurvey data have shown a higher force of infection in poorer neighbourhoods in the municipality of Rio de Janeiro in the 90s [18]–[20]. These studies were conducted 20 years ago in restricted geographical areas; thus, there was a need to estimate the force of infection using the recent prevalence of HAV data for the entire country available in the National Survey. Brazil has undergone improvements in socio-economic conditions in the last two decades. Overall there was a reduction of the Gini coefficient in the Brazilian metropolitan areas from 0.64 (1991) to 0.49 in 2009. The improvement of water supply, sanitation, hygiene and living condition in general has been registered in the country. The change in these key determinants of health status were behind the decrease in infant mortality, diarrhoea disease, and decrease in prevalence of HVA infection [21], [22]. Consequently, the reassessment of HAV strategies is essential. In a previous paper, using data from the urban populations of the Northeast and Midwest macro-regions of Brazil, the age-specific distribution of the population aged 5–19 years that was susceptible to HAV infection was estimated using a simple catalytic model to estimate the force of infection. We showed that HA infection was closely associated with social deprivation, considering individual and contextual factors in a multilevel analysis [12].

This study aimed to identify the transmission pattern of HA infection using a catalytic model based on a primary dataset from the National Hepatitis Survey in a pre-vaccination epidemiological context. We also discuss the methodological issues related to evaluating the impact of the routine implementation of universal childhood HA vaccination at the population level. To our knowledge, this is the first HA transmission model comparing Brazilian macro-regions in the pre-vaccination era.

## Materials and Methods

### Study population and setting

**Figure 1:**
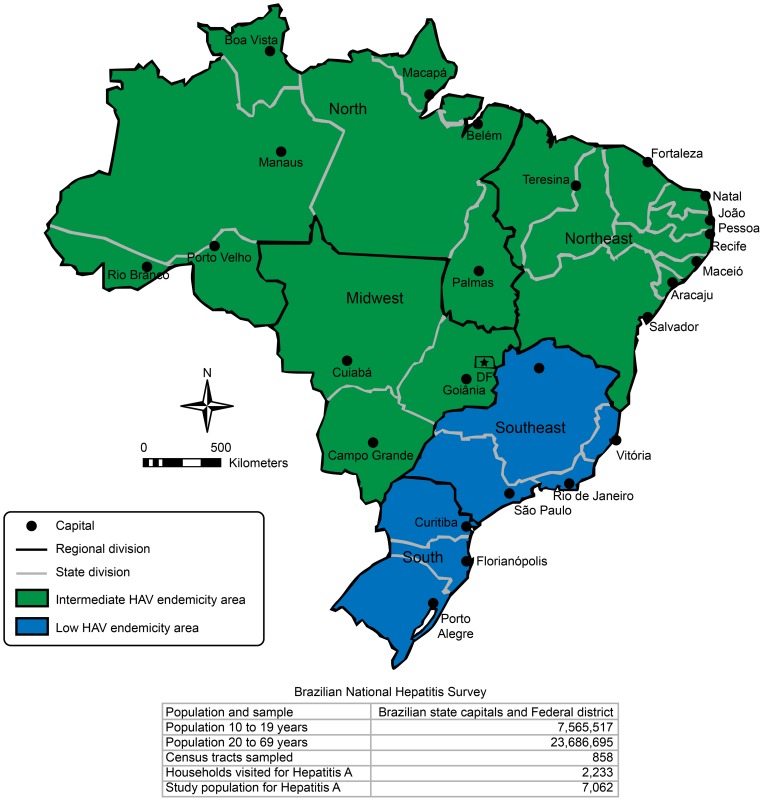
The Brazilian and National Hepatitis Survey populations and the Brazilian macro-regions according to their HA endemicity levels.

The Brazilian population of approximately 190 million individuals is administratively subdivided into 26 states and a Federal District. These administrative units are congregated into five macro-regions (Figure 1). Socioeconomic conditions and sanitary facilities vary greatly across macro-regions, particularly between the more developed macro-regions (South and Southeast) and the less developed macro-regions (North, Northeast, Midwest) of the country [23].

The mathematical model used to estimate the force of infection was built based on primary data of HAV infection from the National Seroprevalence Survey (2005–2009) stratified by age and macro-region [24]. The detailed methodology of this survey has been previously published [25]. Briefly, a household survey aiming to assess the seroprevalence of HAV antibodies among individuals aged 5–19 years was conducted in all 26 Brazilian state capitals and in the Federal District from 2005 to 2009 [12], [25]. A multi-stage cluster sampling scheme was applied based on the Brazilian population as determined by the year 2000 census [23] and stratified by two age groups (5–9 years and 10–19 years). This sampling was considered representative of the population aged 5–19 years in the ensemble of state capitals of each macro-region. The national hepatitis survey was also designed to screen individuals aged 10–69 years for HBV and hepatitis C virus (HCV) antibodies. Figure 1 shows the geographic locations and the population size of the Brazilian macro-regions, the locations of the state capitals and the prevalence of HA in the 5 to 19-year-old age cohort [24].

Of the 6,468 children and young adults screened for anti-HAV antibodies, the estimated seroprevalence ranged from 20% to 30% depending on the macro-region. Two distinct epidemiological patterns emerged: an intermediate endemicity area pattern (North, Northeast and Midwest macro-regions and the Federal District) and a low endemicity area pattern (South and Southeast macro-regions) [12], [24]. The curves of individuals susceptible to HA infection by age were not stable by the age of 19 years that is the curves did not reach the plateau [12]. To extend these curves up to the older age groups, a subgroup of the survey's participants aged 20–69 years (n = 594) from low and intermediate endemicity areas was screened for anti-HAV antibodies, taking advantage of the stored serum samples from the national hepatitis survey. We randomly drew a sample of participants aged 20–69 years old from the intermediate and from the low HAV endemicity areas; the blood samples had been collected as part of the nationwide survey of hepatitis B and C infection. All samples were drawn from urban residents (25).

### Data analysis

#### Descriptive statistics

The estimates of overall prevalence and prevalence by age group and 95% CI were calculated according to the endemicity levels. The frequency of icteric cases among the newly infected individuals was calculated by considering the previously published probabilities of developing jaundice (icteric cases) by age groups (<1 and 1–4 years, 7.20%; 5–9 years, 37.10%; 10–19 years, 70.70%; 20–70 years, 85.20%) [26].

### Estimating the epidemiological parameters

In this model, the seroprevalence data (n = 7,062) from individuals aged 5–69 years residing in intermediate and low endemicity areas were considered.

Let 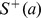 be the proportion of seropositive individuals for HA (according to a positive serological test indicating previous infection) with ages between 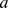 and 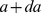. An estimate of the function 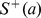 resulted from fitting the serological data to [27]

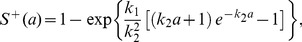
(1)where 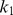 and 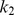 are the fitting parameters estimated by the maximum likelihood method for both regions [19].

In the absence of vaccination, the force of infection 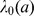 was estimated from the seroprevalence data using the catalytic approach [28] according to the following formula:
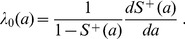
(2)


From equations (1) and (2), we obtain
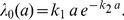
(3)


The seroprevalence data is related to the transmission pattern in the population. To estimate the contact function, 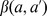, defined as the number of contacts a person with age 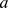 makes with persons with age 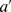 per unit time, we have chosen the following patterns:

1. fully homogeneous mixing model, where the force of infection is the same for all age classes:
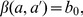
(4)

1. Assortative model
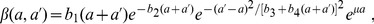
(5)where 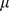 is the mortality rate and 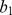, 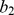, 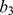 and 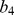 are fitting parameters. This function is the product of a bell-shaped curve for the transversal profile and a positively skewed function that resembles the force of infection given by Equation (3) for the longitudinal profile and is based on the assumption that there is greater interpersonal contact among people of the same age [27], [29].

1. Assortative model plus an additional component, homogeneous by age, accounting for contacts with infected food and water.
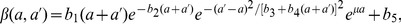
(6)where 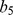 is a constant that gives the infective contact with food and water.

In order to compare the fitting performance of the three models mentioned above we used the Akaike Information Criterion (AIC) [30], calculating the relative likelihood (RL*_i_*) of model *i* as 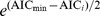, where AIC_min_ is the minimum .of the AIC values for the different models.

We used a Susceptible-Infected-Recovered (SIR) compartmental model [27], [31]. The parameters of the contact functions 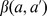 were estimated so that the resulting force of infection, 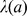, in the absence of vaccination [27],
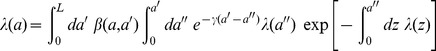



agreed with the 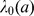 given by Equation (3). To estimate the parameters of 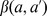 we considered the values of 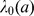 for the 8 age intervals in Table 1.

**Table 1:** The observed prevalence of HA infection from the National Hepatitis Survey, predicted force of infection at the midpoint of the age interval, estimated number of new infections and number of icteric cases stratified by age-group for the intermediate (North-Northeast-Midwest macro-regions) and low (South-Southeast macro-regions) endemicity areas.

	Intermediate endemicity					Low endemicity				
Age group	Prevalence (%)	Force of infection	N° new	Icteric	Population	Prevalence (%)	Force of infection	N° new	Icteric	Population
(years)	(95% CI)	per 100,000 (yr^−1^)	infections	cases		(95%CI)	per 100,000 (yr^−1^)	infections	cases	
<1	-	851	14,580	1,050	1,567,764	-	398	7,393	532	1,516,152
1–4	-	3,699	225,945	16,268	6,396,789	-	1,805	123,178	8,869	6,561,633
5–9	32.9 (22.1; 45.1)	7,823	429,871	159,482	8,123,101	19.8 (18.1; 21.7)	4,249	292,556	108,538	8,796,599
10–14	52.9 (44.2; 61.4)	9,193	343,260	242,685	7,867,220	30.3 (27.8; 32.9)	5,556	315,446	223,020	8,647,387
15–19	63.2 (54.4; 71.4)	9,074	218,771	154,671	8,024,020	43.7 (40.8; 46.7)	6,104	272,118	192,387	8,914,845
20–24	82.2 (67.9; 92.0)	8,225	196,611	167,513	15,931,127	63.4 (46.9; 77.9)	6,158	366,241	312,037	19,151,305
25–29	92.1(78.6; 98.3)	7,088				86.0 (72.1; 94.7)	5,905			
30–39	96.1 (86.5; 99.5)	5,340	51,960	44,270	11,892,064	84.7 (74.3; 92.1)	5,224	152,084	129,576	16,580,832
40–49	95.7 (85.5; 99.5)	3,413	16,737	14,260	9,135,630	89.9 (81.0; 95.5)	4,135	66,869	56,972	14,758,385
50–69	94.1 (83.8; 98.8)	1,595	7,446	6,344	9,873,089	99.2 (95.6; 99.98)	2,664	35,038	29,852	17,371,126
Overall	68.8 (64.8; 72.5)	6,197	1,505,181	806,542	78,810,804	33.7 (32.4; 35.1)	4,304	1,630,923	1,061,785	102,298,264

The derivation of the previous equation comes from the solution of an age-dependent SIR model in the steady-state condition. A more detailed description of both the mathematical derivation of 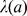 and the numerical solution to estimate the parameters of 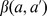 may be found in another publication [27].

Note that, using the procedure described above, the parameters of the contact function are obtained from the seroprevalence data. The fitted contact function may be used, for instance, in models that assess the effects of vaccination programs.

The mortality rate was estimated as the inverse of the life expectancy at birth, taken as 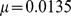 yr^−1^ (life expectancy of 74 years) for the low endemicity areas (South, Southeast) and 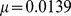 yr^−1^ (life expectancy of 72 years) for the intermediate endemicity areas (North, Northeast, Midwest and Federal District).

The recovery rate 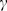 was designated as 8.1 years^−1^, corresponding to an infectious period of 45 days.

From the force of infection, we can define the average age at which susceptible persons acquire infection using the following formula:
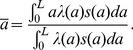
(7)where 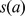, the proportion of remaining susceptible individuals, may be approximated by 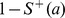.

We have taken the highest age observed in the sero-epidemiological survey (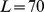 years) as the upper integration limit of the integrals of the equation (7).

The average force of infection was estimated using the definition proposed by Yu et al. [29]

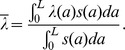



In the steady state preceding vaccination, the proportion of new infections between ages 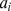 and 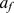 may be calculated using the following formula [32]:
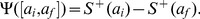
(8)


### Monte Carlo simulations

To compare the seroprevalence fitted curves for the North and South regions and the corresponding force of infection curves, we used Monte Carlo simulations to generate CIs for the curves based on the approach proposed by Amaku et al. [33]. In this case, the Monte Carlo method is a straightforward and accurate alternative for estimating CIs because the fitted parameters for the seroprevalence functions and the corresponding covariance matrices are available. Analytical estimates for the CIs would be more complicated to derive and would provide results with presumably small numerical differences if compared to the Monte Carlo simulations.

The variance-covariance matrix 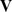 for the seroprevalence function parameters (equation [1]),
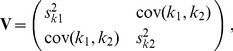
(9)


was estimated from the fitting procedure. In matrix 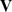, 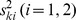 is the variance of parameter 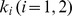, and 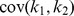 is the covariance between the two parameters.

From the estimated variance-covariance matrix 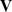, the Monte Carlo algorithm randomly generates 1,000 pairs of values 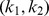 and then calculates CIs for the fitted curves. More details on how this algorithm works may be found in Amaku et al. [33]. We have implemented the computational routines using the R statistical software, version 2.10 (Institute for Statistics and Mathematics, Vienna, Austria) [34].

After estimating the CIs of the seroprevalence curves for the North and South regions, we calculated the ratio between the randomly generated values (within the CIs) for the North and South regions for a given age. If the 100(1-α)% CI for this ratio does not include 1, it means that for that specific age, there is a significant difference between the two compared curves at a level of significance α.

### Ethical issues

Written consent was obtained from all participants or, in the case of minors, from their legal guardians. The project was submitted and approved by the National Research Ethics Committee (CONEP) of the Brazilian National Health Council and by the local research ethics committees at each site.

## Results

**Figure 2:**
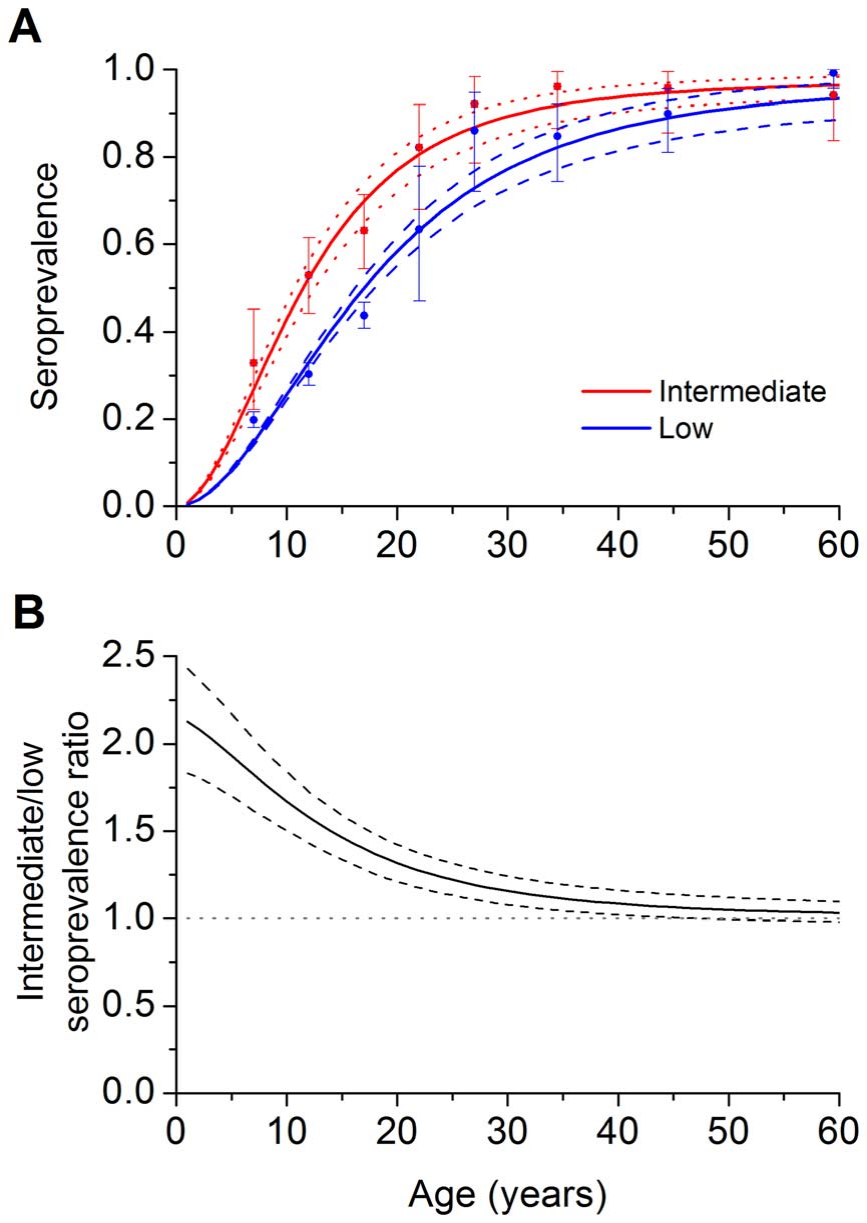
Seroprevalence fitted curves of HA infection (solid lines) for the intermediate (χ^2^ = 6.7, p  = 0.349) and low endemicity (χ^2^ = 85.5, p <0.001) areas (A), the corresponding 99% CIs (red dotted and blue dashed lines, respectively), and 95% confidence intervals for the observed data (error bars); (B) Seroprevalence ratio of HA infection between the intermediate and low endemicity areas by age (solid line) and its corresponding 99% CI (dashed lines). A value of 1 denotes an equal prevalence in both areas, an integer >1 indicates a higher prevalence in the intermediate endemicity area, and a value <1 indicates a higher prevalence in the low endemicity area. The fitting parameters were: 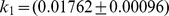 year^−2^ and 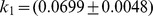 year^−1^ for the intermediate endemicity area and 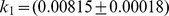 year^−2^ and 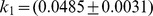 year^−1^ for the low endemicity area.

Figure 2A shows the seroprevalence fitted curves for the North-Northeast-Midwest macro-regions and the Federal District (i.e., intermediate HAV endemicity area) and the South-Southeast macro-regions (i.e., low endemicity area), as well as the corresponding 99% confidence intervals estimated in the Monte Carlo simulations. For both areas, the observed prevalence of HA increased with increasing age, but the curve for the North-Northeast-Midwest macro-region reached the plateau at an earlier age (∼30 years) than the curve for the South and Southeast macro-regions. The ratio between the intermediate and low endemicity seroprevalence curves is shown in Figure 2B. We observed that from the age of 45 years (approximately) onward, the lower limit of the 99% CI includes 1; that is, the estimated prevalence was similar for both endemicity areas.

**Figure 3:**
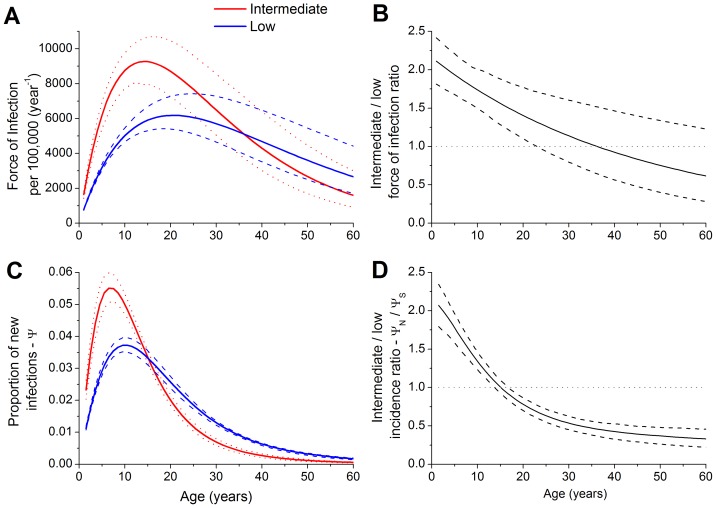
Force of HA infection by age (A), as estimated by the catalytic approach, for the intermediate and low endemicity areas (solid lines) and the corresponding 99% CIs for the curves (red dotted lines for the intermediate endemicity area and blue dashed lines for the low endemicity area). (B) Hepatitis A force of infection curves by age and the ratio between the intermediate and low endemicity areas for the North region (solid line) and its corresponding 99% CI (dashed lines). A value of 1 denotes an equal number of new infected individuals per year in both areas, an integer >1 indicates a higher number of new infected individuals per year in the intermediate endemicity area, and a value <1 indicates a higher number of infected individuals in the low endemicity area. (C) Proportion of new hepatitis A infections for the intermediate and low endemicity regions (red and blue solid lines, respectively) and the corresponding 99% CIs (dotted and dashed lines, respectively). (D) HA incidence ratio (solid line) between the intermediate and low endemicity areas by age and its 99% CI (dashed lines).

The mean age of infection was 13.8 years (95% CI, 12.8–14.7) in the intermediate endemicity areas and 19.4 years (95% CI, 18.7–19.9) in the low endemicity areas. The force of infection curves, predicted using the catalytic approach defined in equations (2) and (3), are shown in Figure 3A with their corresponding 99% CIs. There is an overlap between the CIs for ages older than 20 years when the lower limit of the 99% CI for the force of infection ratio becomes lower than 1 (Figure 3B). Figure 3C depicts the estimates for the proportion of new infections (equation [6]) in the absence of vaccinations for the North-Northeast-Midwest and South-Southeast macro-region considering 1-year age intervals in the equation (8). In Figure 3D, we show the corresponding incidence ratio, which is <1 from the age of 15 years, indicating that the predicted incidence was higher at an earlier age in the North-Northeast-Midwest macro-regions than in the South-Southeast macro-regions.

**Figure 4:**
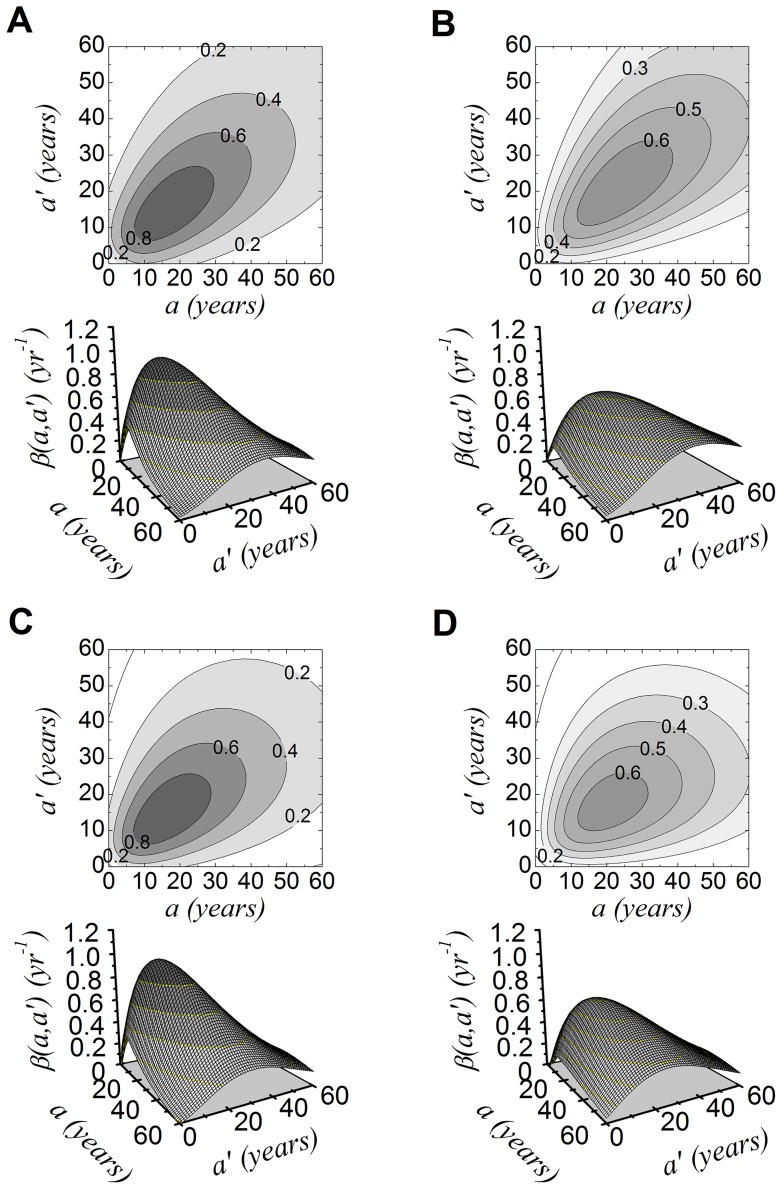
The contact function 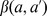 and the respective contour plot for the intermediate (A, assortative; C, assortative with food and water transmission) and low (B, assortative; D, assortative with food and water transmission) endemicity areas. The fitted parameters were: (A) 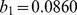 year^−2^, 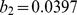 year^−1^, 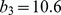 year and 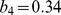; (B) 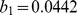 year^−2^, 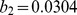 year^−1^, 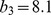 year and 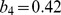; (C) 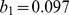 year^−2^, 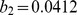 year^−1^, 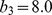 year, 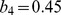 and 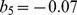 year^−1^; (D) 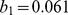 year^−2^, 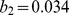 year^−1^, 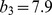 year, 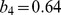 and 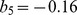 year^−1^; for the fully homogeneous model (not shown), 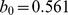 year^−1^ (intermediate) and 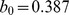 year^−1^ (low).

The predicted contact functions, 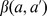, for the intermediate and low endemicity areas are shown in Figure 4. Note that the intensity of transmission is higher in the intermediate endemicity areas. It should be highlighted that differences between models are hardly noticed by visual inspection.

Table 1 summarises the observed prevalence of HA infection, the predicted force of infection, the predicted number of newly infected individuals and the estimated number of icteric cases by age group and stratified by endemicity levels. The overall age-adjusted HA prevalence was 68.8% (95% CI, 64.8%–72.5%) and 33.7% (95% CI, 32.4%–35.1%) in the intermediate and low endemicity areas, respectively. In the intermediate endemicity area the prevalence was 32.9% in the 5–9 years old age group and in the low endemicity it was 19.88% in the same age-group. In the intermediate endemicity areas, the peak force of infection was identified in the 10- to 19-year-old age group (∼9,000 infected individuals per year per 100,000 susceptible persons), whereas the peak force of infection occurred in the 15- to 29-year-old age group (∼6,000 infected individuals per year per 100,000 susceptible persons) for the other macro-regions. The estimated number of icteric cases was 806,452 in the intermediate HAV endemicity areas, of which 52% were younger than 15 years old, while in the low endemicity areas, the estimated number of icteric cases was 1,061,785, of which 32% were younger than 15 years old.

The age at midpoint of population susceptibility, defined as the age at which half of the population in that age group does not have anti-HAV IgG antibodies, was 11.5 and 17 years, respectively, for the intermediate and low HAV endemicity areas.

Table 2 shows the results of AIC for the three contact functions. It can be noticed that assortative model has the minimum AIC value and therefore is the model that minimizes the estimated information loss.

**Table 2:** Comparison of three models of contact function (equations 4, 5 and 6) by the Akaike Information Criterion (AIC) and the relative likelihood (RL) of the models.

		Low Endemicity		Intermediate Endemicity	
Model	Parameters	AIC	RL	AIC	RL
Fully homogeneous	1	142.268	1.12E-26	281.491	6.56E-57
Assortative	4	22.759	1	22.757	1
Assortative + food/water transmission	5	24.705	0.378	24.707	0.377

## Discussion

The mathematical modelling approach used in this work, based on a SIR epidemic model together with a Monte Carlo method to estimate confidence intervals for the age-dependent curves, allowed for the comparison of the rates of HA infection by age according to the two endemicity levels in Brazil. In the more socioeconomically developed macroregion (South and Southeast), considered low HAV endemicity, the age of virus exposure occurred later compared to the intermediate endemicity areas. At least half of the population aged 10- to 14-years-old and older was still susceptible to HA infection in the intermediate and low endemicity areas. The model herein predicted a gradual build-up of new HA infections during adolescence and adulthood but with different force of infection in both endemic settings.

A comprehensive HA survey conducted in Brazil from 2005 through 2009 showed a lower prevalence (∼40%) among children and adolescents [24] than previously reported in selected areas [35]–[37]. In our preliminary modelling approach, the fitted curves of prevalence using a dataset comprising individuals aged 5–19 years from this national survey still presented an ascending trend of infection in the second decade of life. Therefore, we extended the detection of specific IgG serology for a subsample of the adult population to build a full mathematical model that encompassed all age strata. At the population level, the observed prevalence of HA infection was highest at 30–39 years, as shown by the saturation of the infection levels in the intermediate endemicity area. Conversely, the highest observed prevalence was achieved two decades later (50–59 years) in the low endemicity setting. The prevalence rates were statistically higher in the intermediate endemicity areas until the age of 45 years compared to the low endemicity areas but similar in both areas at older ages, as depicted by the prevalence ratio curves.

The two models, built according to the endemicity of the Brazilian regions, yielded important information about the difference in the force of HA infection curves by age. First, the transmission curves showed similar shapes in both areas, skewed to the right toward the young age groups. The risk of acquiring HA infection was higher for children and adolescents until the age of 25 in the intermediate endemicity region, as depicted by the ratio of the force of infection curves between regions. Our findings seem compatible with a previous mathematical model of the USA population, which used data from a national population serosurvey and estimated differences in the force of HA infection between age groups across the different regions [17].

Although data-driven age-specific contact pattern were shown to be an alternative route to the estimation of epidemiological parameters [38], [39], specific contact data for the two Brazilian macro-regions are not available because no similar studies were undertaken in Brazil.

The HA models presented herein, using the assortative model for the contact function, were incorporated into a cost-effectiveness analysis of HA vaccination for Brazil [40]. When using these parameters, universal HA vaccination was shown to be cost-saving in both endemic settings. We are aware that the presented model simplifies HA epidemiology by primarily focusing on direct transmission of the infection and excludes the issues related to outbreaks and population movements between areas. Another possible limitation of the analysis is using the serosurvey data from an urban population that did not include rural and institutionalised populations. Nevertheless, these models used seroprevalence data from a representative sample of the Brazilian urban population, which accounts for 84% of the total population of the country [41]. Additionally, the force of infection by age group could be considered a baseline transmission parameter for the two endemicity regions.

Assuming the potential upcoming addition of the HA vaccine to the routine immunisation schedule in Brazil, there is a need to discuss the best methodological approach for evaluating the impact of this intervention at the population level [42]. In general, a decrease in the incidence of disease, as reported by surveillance systems, is used to evaluate the protective effect of vaccination [43]. For example, in southern Italy, age-stratified case notification analysis showed a reduction in HA case notification when comparing the HA pre-vaccination and post-vaccination implementation periods [44], [45]. However, the usefulness of this design depends on the quality of the HA surveillance system. In Brazil, HA cases are generally underreported, and not all cases are laboratory confirmed or investigated [46]. At least two issues should be considered in evaluating HA vaccination. First, asymptomatic infections may go undetected if case notification is the outcome measure. Second, the serological marker of infection is the same as the HA vaccine. One alternative would be to design repeated serosurveys of unvaccinated people to determine the age distribution pattern of HA infection, as is generally recommended to evaluate the immune status of a population [20], [47]. Therefore, the force of infection could be obtained by modelling the prevalence data, which can show changes in the force of infection trends over time after vaccination implementation. This same strategy of using studies on the force of infection trends derived from serosurveys was useful for evaluating the impact of measles vaccination [48]. In addition, a serosurvey that includes a vaccinated cohort would measure vaccination coverage and the herd immunity effect at the population level. The temporal trend of fatal HA cases and liver transplantation resulting from HA infection should be monitored in the post-vaccination era to measure the burden of the disease, as recommended by a WHO position paper [1]. In addition to evaluating HA vaccination implementation, studies should take into account the potential reduction of viral transmission over time because of improvements in socioeconomic conditions. In Brazil, the HA vaccination evaluation should encompass the two diverse transmission patterns identified by our study regardless of the study design adopted.

Our study differs from other previous HA incidence models in Brazil in that it (1) uses a large dataset that is representative of urban populations and various age groups, (2) compares two different endemicity levels, (3) has the ability to distinguish diverse transmission patterns according to endemicity levels, (4) demonstrates striking differences in the force of infection related to age, as depicted by the ratio curves between the endemicity levels and (5) demonstrates the increasing number of susceptible individuals and, consequently, the increase in incidence in adults

Finally, our findings support the shift of Brazil toward intermediate and low endemicity levels with the shift of the risk of infection to older age groups. We believe that these findings of HA force of infection stratified by age and endemicity levels might be useful as baseline data in a pre-vaccination scenario for Brazil.
